# Decoding the Genetic Basis of Salinity Tolerance at Germination and Seedling Traits in HEB-25 Barley NAM Population

**DOI:** 10.3390/plants15121886

**Published:** 2026-06-17

**Authors:** Radwa Y. Helmi, Mohammed A. Sayed, Abdelhadi A. Abdelhadi, Andreas Maurer, Andreas Börner, Nagwa I. Elarabi, Asmaa A. Halema, Matías Schierenbeck, Mahmoud M. Sakr, Klaus Pillen, Helmy M. Youssef

**Affiliations:** 1 Genetics and Cytology Department, Biotechnology Research Institute, National Research Centre, Giza 12622, Egypt; youssefr@ipk-gatersleben.de; 2 Leibniz Institute of Plant Genetics and Crop Plant Research (IPK), Corrensstr. 3, OT Gatersleben, D-06466 Seeland, Germany; boerner@ipk-gatersleben.de (A.B.); schierenbeck@ipk-gatersleben.de (M.S.); 3 Agronomy Department, Faculty of Agriculture, Assiut University, Assiut 71526, Egypt; msayed@aun.edu.eg; 4 Department of Genetics, Faculty of Agriculture, Cairo University, Giza 12613, Egypt; abdelhadi.abdallah@agr.cu.edu.eg (A.A.A.); nagwa.abdulfattah@agr.cu.edu.eg (N.I.E.); assma.ahmed@agr.cu.edu.eg (A.A.H.); 5 Chair of Plant Breeding, Institute of Agricultural and Nutritional Sciences, Martin-Luther-University Halle-Wittenberg, Betty-Heimann-Str. 3, D-06120 Halle (Saale), Germanyklaus.pillen@landw.uni-halle.de (K.P.); 6 Plant Biotechnology Department, Biotechnology Research Institute, National Research Centre, Giza 12622, Egypt; msakr@asrt.sci.eg; 7 Center for Converging Sciences and Emerging Technologies (CoSET), Benha National University, Al Obour 13518, Egypt; 8 Department of Botany, Faculty of Agriculture, Cairo University, Giza 12613, Egypt

**Keywords:** barley, GWAS, salinity, SNPs, QTLs, gene annotation

## Abstract

Climate change is intensifying soil salinization, posing a major threat to crop establishment and productivity, particularly in arid and semi-arid regions. Barley (*Hordeum vulgare* L.), one of the most salt-tolerant cereals, offers valuable genetic resources for improving salinity resilience at early growth stages. This study exploited the genetic diversity of the Nested Association Mapping (NAM) population Halle Exotic Barley-25 (HEB-25) to dissect salinity tolerance during germination and seedling developmental stages. First, the HEB-25 parental lines (25 wild barley genotypes and cv. Barke) were evaluated under salinity treatment to identify contrasting responses. Based on this screening, four HEB families (01, 04, 09, and 22) were selected out of 25 HEB families for detailed phenotypic and genomic analysis. Seeds of the selected HEB families were subjected to 40% seawater salinity stress and control treatments to assess germination percentage and seedling traits, including shoot length, root length, fresh weight (FW), dry weight (DW), DW/FW ratio, root–shoot ratio, and salt tolerance index (STI). Substantial variation was observed among families for all measured traits under salinity stress. STI values enabled clear differentiation among families: Family 01 exhibited the most consistent overall tolerance profile, Family 22 showed the strongest sensitivity in biomass traits, and Family 04 displayed a trait-specific response with sensitivity at the family-mean level but exceptional within-family diversity, harboring some of the highest individual TI values across the population. A genome-wide association study was conducted using 32,995 SNP markers. A total of 27 significant SNPs were identified, corresponding to 20 quantitative trait loci (QTLs). Of these, 12 QTLs were detected under control conditions, 16 under seawater treatment, and 21 based on tolerance indices, indicating both constitutive and stress-responsive genetic effects. Gene annotation within these regions revealed approximately 23 candidate genes associated with abiotic stress tolerance, including genes involved in ion transport, osmotic adjustment, kinases and stress signaling pathways. HEB_22_003, HEB_04_087, and HEB_01_013 represent the most promising genotypes for salinity breeding. These findings highlight the effectiveness of combining precise phenotyping with high-resolution genomic analysis in the HEB-25 population to uncover the genetic architecture of salinity tolerance at early developmental stages. We identified 20 salinity-responsive QTLs, including five major-effect loci on chromosomes 2H, 4H, 5H, and 7H that consistently explained the largest share of phenotypic variation. These loci co-localized with candidate genes linked to ion homeostasis, Ca^2+^-mediated signaling, protein glycosylation, epigenetic regulation, and root system plasticity, revealing key mechanisms underlying early-stage salt adaptation in barley. The strong and contrasting responses of Family 01 and Family 04 provide an excellent genetic framework for functional validation of tolerance alleles. Collectively, these genomic resources establish a robust foundation for QTL pyramiding, marker-assisted breeding, and the development of climate-resilient barley cultivars for saline agroecosystems.

## 1. Introduction

Barley (*Hordeum vulgare* L.) is one of the major cereal crops worldwide and is recognized for its remarkable adaptability to diverse and extreme environments [1,2]. It is also considered more salt-tolerant than other cereals and has frequently served as a model species for studying salinity tolerance mechanisms applicable to other crops [3,4]. Egypt is among the countries most severely affected by soil salinity, with approximately 35% of its agricultural lands classified as salt-affected, particularly in newly reclaimed areas and coastal zones where groundwater irrigation further exacerbates salinization [5]. Barley cultivation in Egypt is predominantly concentrated in rainfed and newly reclaimed lands, where limited water supply and high soil salinity represent major constraints to productivity, making salinity tolerance a critical breeding target [6]. However, intensive selection and breeding over the last centuries have narrowed the genetic diversity of cultivated barley compared to its wild ancestor, *Hordeum vulgare* ssp. *spontaneum*, reducing its adaptive potential under stress conditions [2,7,8].

Climate change is a major driver of abiotic stress, significantly affecting agricultural land and limiting plant growth and productivity [9,10]. Among abiotic stresses, soil salinity is one of the most severe constraints to crop production worldwide. Approximately 20% of cultivated land and 33% of irrigated land are affected by salinity, with 1–2 million hectares becoming salinized each year [11]. Salinity reduces crop yield primarily through two mechanisms: (i) osmotic stress, which limits water and nutrient uptake, and (ii) ionic toxicity caused by excessive accumulation of Na^+^ and Cl^−^ ions, leading to metabolic imbalance and impaired physiological processes [12,13].

The impact of salinity is strongly stage-dependent, with germination and early seedling development being considerably more sensitive than later growth stages [14]. Stress at these early stages can irreversibly affect plant establishment and ultimately reduce yield potential [15]. Therefore, improving salinity tolerance during germination and seedling growth is a critical target for crop improvement, particularly in regions where dry summers increase salt accumulation in the topsoil [16]. Several agronomic strategies have been proposed to mitigate salinity effects, including improved irrigation management, soil leaching, drainage enhancement, and the use of partly desalinated water. However, the most economical and sustainable approach remains the development of salt-tolerant cultivars [17,18]. Achieving this requires a comprehensive understanding of the physiological and genetic mechanisms underlying salinity tolerance across developmental stages [19].

Salinity tolerance in barley is a complex quantitative trait governed by multiple quantitative trait loci (QTLs) and genes involved in ion homeostasis, osmotic adjustment, and stress signaling [4,20]. Major QTLs have been mapped on chromosomes 2H, 4H, and 7H, with a prominent locus on 7H associated with shoot Na^+^ exclusion [21,22]. Functional studies have identified key genes, such as *HvHKT1;5* on chromosome 4H, which mediates Na^+^ retrieval from the xylem [23], and *HvNHX1*, a vacuolar Na^+^/H^+^ antiporter that contributes to intracellular ion compartmentalization [24]. Additional components include the SOS pathway (*HvSOS1*) and transcription factors such as *HvDREB1* and *HvWRKY* that regulate stress-responsive gene expression [25]. Integrative approaches combining QTL mapping, genome-wide association studies (GWAS), and transcriptomics have further advanced the understanding of the genetic architecture underlying salinity tolerance [26]. Despite these advances, limited attention has been given to the genetic basis of salinity tolerance during germination and early seedling stages under realistic saline conditions. Most previous studies simulated salinity using NaCl solutions alone [4,27], which do not fully represent the complex ionic composition of natural saline soils or seawater irrigation. Using seawater provides a more ecologically relevant and ionically complex stress environment.

To reintroduce lost diversity from wild barley into elite backgrounds, the nested association mapping (NAM) population Halle Exotic Barley-25 (HEB-25) was developed by crossing 25 wild barley accessions with an elite cultivar (cv. Bark) [28]. This population has been widely used to dissect complex traits, including flowering time, yield, drought tolerance, grain quality, and disease resistance [29,30,31,32,33,34,35,36].

In this study, the parental genotypes of the HEB-25 population (Barke and 25 wild barley accessions), as well as 185 selected easy-to-handle BC1S3 genotypes from four selected families (01, 04, 09, and 22), were evaluated to investigate salinity tolerance during germination and seedling developmental stages under diluted seawater treatment. Using high-density SNP genotyping and GWAS, we aimed to (i.) compare the response of the four families to salinity stress, (ii.) identify QTLs, marker–trait associations, and candidate genes associated with early-stage salinity tolerance for each family and for the combined analysis. The use of the HEB population enables evaluation of wild-allele introgressions within an elite background, providing a powerful system to dissect the genetic basis of salinity adaptation during critical early growth stages.

## 2. Results

### 2.1. Growth Assessment of Parental Genotypes of the HEB-25 Population

As shown in Table S1, the summary statistics demonstrate a significant inhibitory effect of salinity treatment on plant growth at the seedling stage, particularly evidenced by the dramatic decline in SVI and the 60% reduction in ShL, which identifies shoot elongation as a highly sensitive stage. While Ger% remained relatively stable, the subsequent growth phase suffered, though an adaptive shift was observed in the RSR, which increased to 1.6 with high phenotypic variation (C.V. = 40.5%). Furthermore, the decrease in WC% alongside stable SDW values suggests that the reduction in seedling fresh weight is primarily driven by osmotic water loss rather than a failure in dry matter accumulation.

In Table S2, the mean squares (MS) for all studied traits showed highly significant differences among the parental genotypes of the HEB-25 population, confirming notable genetic variation across both control and salt-stress environments. The raw data are given in Table S1. The coefficient of determination (R^2^) is generally high (mostly > 0.80), indicating that the experimental model explains a large portion of the observed variation. In addition, the broad-sense heritability (H2) values are remarkably high for most traits, often exceeding 90%. This suggests that the phenotypic variance is largely determined by genetic factors rather than environmental noise, which is an excellent indicator for successful selection in breeding programs. The highest heritability was recorded for Ger% (95.5%) and RL (96.3%) under salinity, suggesting these traits are strongly genetically fixed. In contrast, the lowest heritability was observed in RSR (58.4%) under salinity stress, while it was 89.9% under control conditions, indicating that the RSR is more sensitive to environmental fluctuations when salt stress is applied.

### 2.2. Evaluation of Salt Tolerance Indices (STIs) of the Parental Genotypes

The analysis of salt tolerance indices (STIs) reveals substantial genetic diversity among the genotypes, as evidenced by the significant mean squares (MS) across all parameters (Table S3). High broad-sense heritability for Ger%TI (90.6%) and ShLTI (92.6%) indicates that these traits are primarily governed by genetic factors. In contrast, the SDWTI exhibited the lowest heritability (15.3%) and model fitness (R^2^ = 0.36), suggesting that dry matter accumulation is more susceptible to environmental factors. Finally, the narrow C.V.% and high mean of WCTI (92.7%) reflect a relatively uniform capacity for water retention across the population.

Based on the comprehensive growth assessment and the evaluation of the five salinity tolerance indices for the parental genotypes of the HEB-25 population, specifically, the superior performance in biomass retention and germination stability, families no. 01, 04, 09, and 22 were identified as the most salt-tolerant ones (Figure S1). These four families consistently outperformed the elite cultivar ‘Barke’ and other accessions across multiple growth parameters, showing highly significant tolerance levels.

### 2.3. Phenotypic Variation Among Barley Genotypes of the Four Selected Families

In total, 185 barley genotypes were evaluated for salinity tolerance under control and 40% seawater treatment. Our results revealed substantial phenotypic variation for the measured traits within and among the genotypes of the four families (Table S2). The ANOVA results showed that genotype, treatment and their interaction differed significantly at *p* < 0.01. The analysis of variance for germination and seedling growth parameters showed significant effects of genotypes, treatments and genotypes x treatment interactions for the four families. The results showed extensive genotype variation in the majority of the studied traits, like DW, FW, DW/FW_R, RL, RSR and ShL. Moreover, there was substantial phenotypic variation for most of the measured traits (Table 1). For example, the mean shoot length under control (ShLC) and root length under control (RLC) were 14.936 and 14.465 cm, respectively, whereas under 40% seawater stress these values reduced to 4.788 and 5.139 cm. The coefficient of variation (CV) values increased under salinity conditions for the majority of the traits studied as compared to the control, except for FW and DW, which decreased under salinity conditions (Table 1). The variations observed in the barley genotypes are sufficiently diverse to show different responses under 40% salinity stress (Table 1).

**Figure 1:**
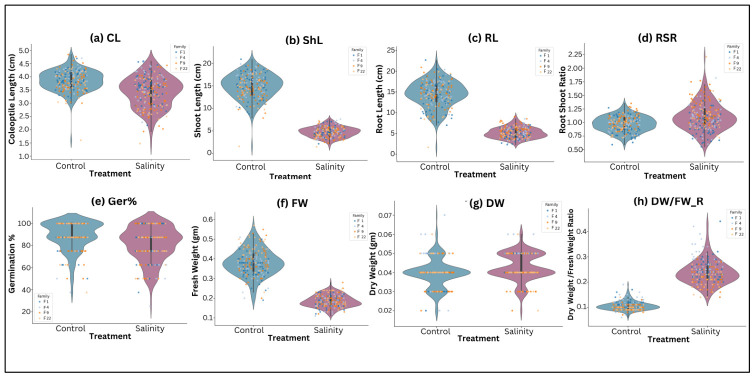
Comparative phenotypic response to salinity stress. (**a**) Coleoptile length (CL); (**b**) Shoot length (ShL); (**c**) Root length (RL); (**d**) Root Shoot Ratio (RSR); (**e**) Germination percentage (Ger%); (**f**) Fresh weight (FW); (**g**) Dry weight (DW); (**h**) Dry Weight/Fresh Weight Ratio (DW/FW_R).

The results displayed no significant reductions between the genotypes under seawater treatment compared with the control for the majority of the traits, including coleoptile length (CL), RSR and Ger% (Figure 1). In contrast, decreases were observed in shoot length, root length, fresh weight and dry weight under salinity treatment compared with the control. While the results showed a higher DW/FW ratio under salinity treatment than in the control.

### 2.4. Evaluation of Salt Tolerance Indices (STIs) of Four Selected Families

**Figure 2:**
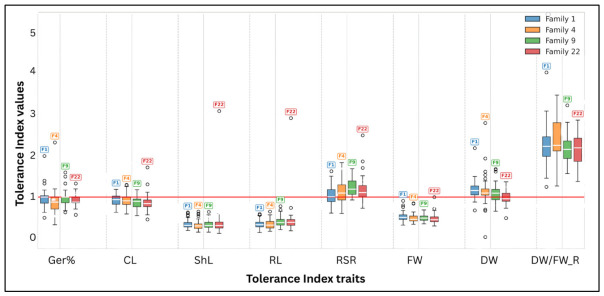
The tolerance index for the four families (F 1 (56 genotypes), F 4 (42 genotypes), F 9 (52 genotypes), and F 22 (35 genotypes)) under 40% seawater stress. The degree of significance for all correlations was *p* ≤ 0.001. The red horizontal line indicates a tolerance index value of 1.0, representing the threshold between salt-sensitive (TI < 1) and salt-tolerant (TI > 1) genotypes.

Significant variations were observed among HEB 01, 04, 09 and 22 families at the seedling growth tolerance index (TI), with differences detected across all traits (Figure 2 and Table S5).

Family 01 demonstrated the most consistent overall tolerance profile, showing higher TI values for CL, DW, FW, and Ger%, reflecting stable biomass accumulation and germination maintenance under saline conditions. In contrast, family 22 showed the lowest TI values for FW, DW, and DW/FW-R, recommending greater sensitivity in biomass-related traits, while family 04 exhibited the lowest family-level TI means for Ger%, ShL and RL, reflecting impaired early establishment and shoot/root elongation under salinity stress.

Analyses of family 01 genotypes revealed considerable variation in TI means across genotypes for all traits (Table S5). Among these genotypes, HEB01-013 revealed the highest value of DWTI (2.19) and RLTI (0.59), HEB01-031 for ShLTI (0.63), HEB01-080 for RSRTI (1.63), HEB01-101 for FWTI (0.91), and HEB01-133 for DW/FW_RTI (4.03), suggesting higher salinity stress tolerance. In contrast, genotypes such as HEB01-003 displayed the lowest values of ShLTI (0.21) and DWTI (0.68), HEB01-075 for ShLTI (0.21), HEB01-101 for DW/FW_RTI (1.25), HEB01-133 for RLTI (0.14) and FWTI (0.32) and HEB01-134 for RSRTI (0.06), indicating reduced tolerance.

Similarly, analyses of family 04 genotypes revealed substantial within-family variation (Table S5). Although family 04 harbored genotypes with notably high individual TI values, including HEB04-087 with the highest DWTI (2.80) and DW/FW_RTI (5.61), HEB04-062 for CLTI (1.30), HEB04-070 for RSRTI (1.84), HEB04-080 for RLTI (0.65), HEB04-136 for FWTI (0.85), and HEB04-1499 for ShLTI (0.65), this family simultaneously showed the lowest family-level TI means for Ger%, ShL and RL, indicating trait-specific rather than comprehensive tolerance. Sensitive genotypes within this family included HEB04-078 with the lowest DWTI (0.03), HEB04-063 for RLTI (0.17), HEB04-122 for ShLTI (0.15) and FWTI (0.34) and HEB04-017 for CLTI (0.64).

For family 09, specific genotypes demonstrated superior seawater tolerance as evidenced by high TI values (Table S5). Notably, HEB09-005 recorded the highest DWTI (1.68), HEB09-039 peaked for RLTI (0.78), HEB09-064 for ShLTI (0.64), HEB09-072 for DW/FW_RTI (3.23), HEB09-089 for RSRTI (1.69) and HEB09-164 for CLTI (1.18). Conversely, sensitive genotypes included HEB09-049 with the lowest FWTI (0.35), HEB09-084 for both FWTI (0.35) and DWTI (0.66), HEB09-110 for RLTI (0.21) and HEB09-140 for ShLTI (0.15).

For family 22, high-performing genotypes included HEB22-003 for CLTI (1.72), HEB22-008 for DW/FW_RTI (2.78), HEB22-053 for RSRTI (2.50), HEB22-109 for FWTI (1.00) and DWTI (1.37) and HEB22-114, which achieved the highest TI for both ShLTI (0.60) and RLTI (0.74), collectively suggesting a robust capacity for salinity stress adaptation (Table S5). In contrast, minimum TI values were recorded for HEB22-023 for FWTI (0.30) and DWTI (0.49), HEB22-053 for CLTI (0.46) and ShLTI (0.12), HEB22-103 for RSRTI (0.73), HEB22-109 for DW/FW_RTI (1.38) and HEB22-128 for RLTI (0.18), indicating sensitivity in these genotypes.

The four families exhibited adaptive responses in CL, RSR, Ger%, and DW/FW-R under 40% seawater stress, as shown in Figure S2. Family 01, 09, and 22 genotypes maintained or increased DW while showing reductions in ShL, RL, and FW compared to control conditions (Figure S2A,C,D). In contrast, family 04 showed a significant decrease in DW as well (Figure S2B), further distinguishing it from the other families and reinforcing its classification as the most sensitive family in terms of overall biomass response under salinity stress.

### 2.5. Correlation Between Phenotypic Traits

**Figure 3:**
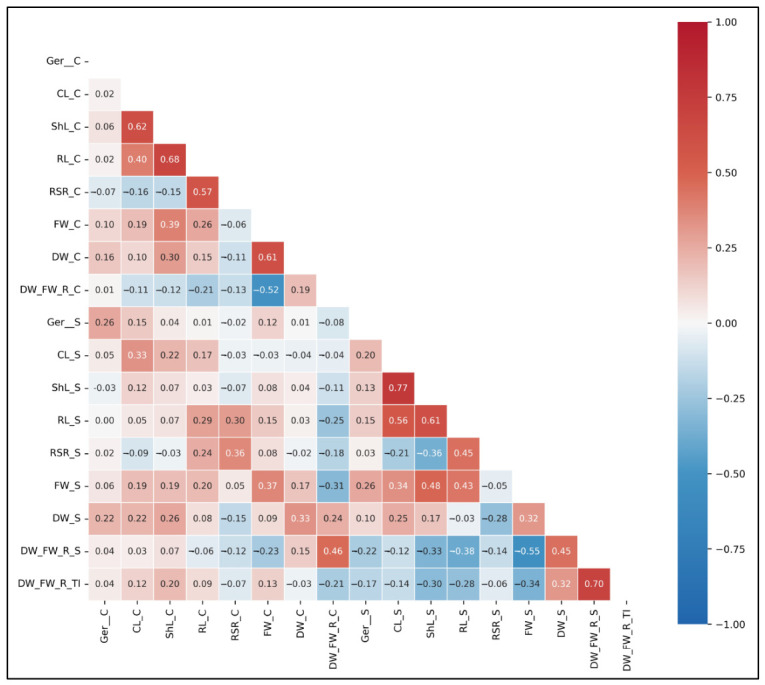
Phenotypic Correlation Heatmap including 185 genotypes derived from four HEB-25 Families. The degree of significance for all correlations was *p* ≤ 0.001. The color reflects the strength of the correlation.

The correlations among all families reveled positive correlation between CLS and SHLS (r = 0.77) and a negative correlation between DW/FW_RS and FWS (r = −0.55) (Figure 3).

A highly positive correlation was displayed between DW/FW_RS and DW/FW_RTI (r = 0.82), CLS and ShLS (r = 0.83), CLS and ShLS (r = 0.86) and DR/FW_RS with DW/FW_RTI (r = 0.0.89) for families 01, 04, 09 and 22, respectively. Furthermore, a negative correlation was observed (r = −0.62) between both DW/FW_RS and DW/FW_RTI with FWS and ShLS, respectively, for family 01 (Figure S3). Negative correlations (r = −0.64) were detected between ShLS and DW/FW_RS for family 04 (Figure S4). For families 09 and 22, negative correlations were also found between DW/FW_RC and FWC (r =−0.59) and between ShLS and RSRS (−0.53), respectively (Figures S5 and S6).

### 2.6. Multivariate Analysis: Principal Component Analysis (PCA) and Clustering

**Figure 4:**
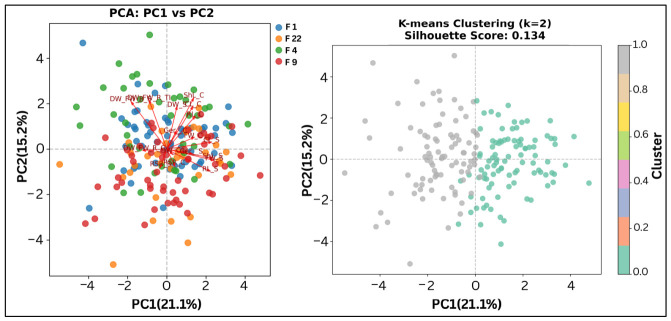
Principal Component Analysis (PCA) biplot of 185 lines from four HEB-25 families (01, 04, 09 and 22) evaluated under salinity stress conditions. The two principal components (PC1 and PC2) explained the largest proportion of total phenotypic variation among genotypes. Dots represent individual genotypes and are color-coded by family: family 01 (blue), family 04 (green), family 09 (red), and family 22 (orange). Solid lines (loading vectors) represent the eight measured traits for control and salinity. The direction and length of each vector indicate the contribution and correlation of each trait with the principal components. The right panel shows K-means clustering (k = 2) of the same genotypes, with a Silhouette Score of 0.134, indicating overlapping clusters with limited separation between the two groups.

To investigate the relationships among trait variables and the factors underlying trait variation, PCA was performed for all eight traits. PCA results revealed that each PC can only explain a small variance, such as PC1 explaining 18.2, 19.5, 15.8 and 19.9% for families 01, 04, 09 and 22, respectively, and PC2 explaining 25.0, 25.2, 29.0 and 25.5% of total variance for families 01, 04, 09 and 22, respectively (Figure S7). Condensed PCA included eight traits, which explained 36.3% of total variance (Figure 4). The PCA-based clustering classified the family 01 genotypes into two distinct groups, yielding a Silhouette score of 0.150 [37,38], explaining 43.2% of the total variance. Similar patterns were observed for families 04 and 09, which achieved Silhouette scores of 0.151 and 0.164, respectively. In contrast, the data for family 22 were divided into three groups, resulting in a Silhouette score of 0.153 and an explained variance of 45.4% across PC1 and PC2 (Figure S6). To further elucidate the relationships between measured traits and genotypes under seawater for all families together, PCA was conducted for both salinity and control treatments. The data divided the genotypes into two groups with a Silhouette score of 0.134 and an explained variance of 36.3% across PC1 and PC2 (Figure 4). All Silhouette scores ranged between 0.130 and 0.164, indicating weak cluster separation and considerable overlap between groups.

### 2.7. GWAS Analysis

**Figure 5:**
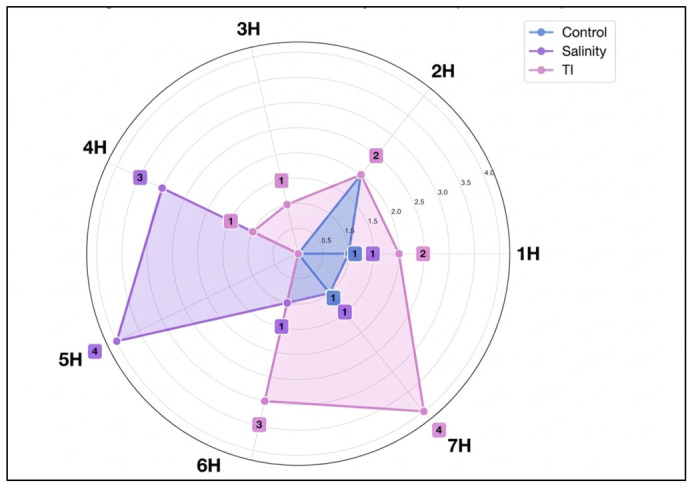
Distribution of detected QTLs across the barley genome in the four HEB-25 families combined under control and salinity conditions, and for the Tolerance Index (TI). QTLs identified under control conditions are shown in blue, those detected under salinity stress in purple, and TI-associated QTLs in pink.

The salinity tolerance data were combined with pre-existing SNP data [39] to carry out a genome-wide association study and to locate marker–trait associations (MTA) based on the set of 185 HEB lines from the four HEB families 01, 04, 09 and 22. The analysis revealed a total of 27 significant MTAs for control, salinity and tolerance indices. Their distribution on the barley chromosomes was diverse, as shown in Figure 5.

The GWAS analysis across all genotypes identified 20 QTLs related to germination and different seedling growth parameters under control (QTLs), seawater treatment (QTLs) and their tolerance indices (QTLs). These QTLs were unevenly distributed across the seven barley chromosomes, as provided in Table 2. Chromosome 7H emerged as the most QTL-rich chromosome (5 QTLs), indicating stable loci shared across genetic backgrounds, followed by chromosomes 1H, 2H, 4H, 5H and 6H (3 QTLs each), whereas 1 QTL was detected on chromosome 3H.

**Figure 6:**
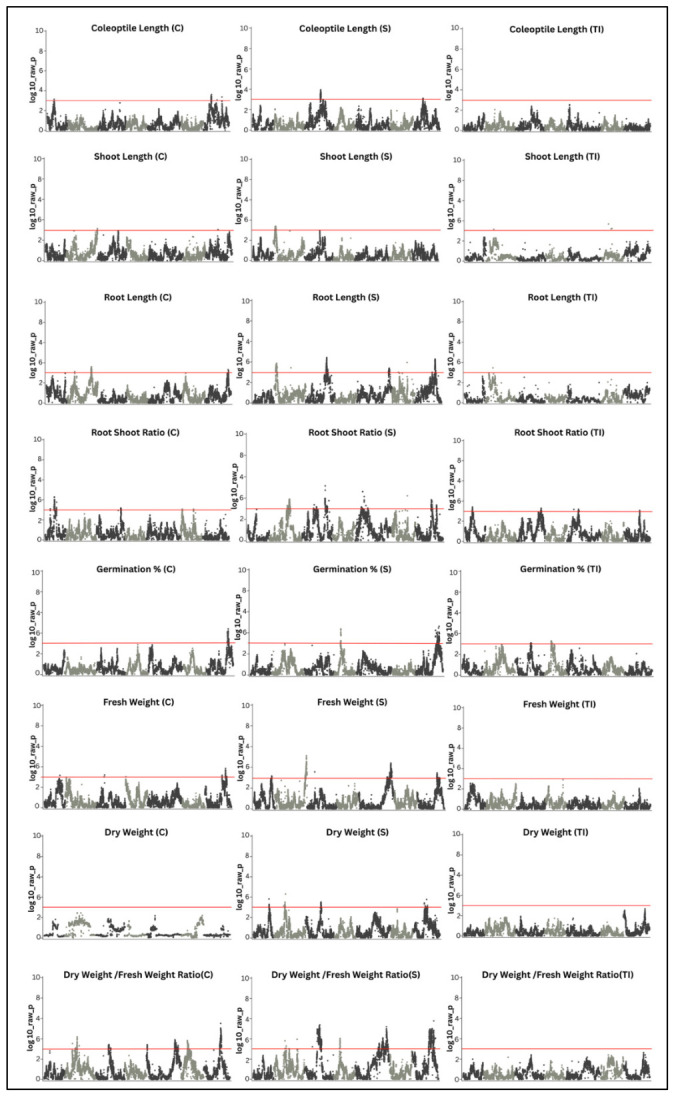
Manhattan plot showing marker–trait associations calculated across four HEB-25 families. The horizontal axis represents the seven barley chromosomes (1H–7H), and the vertical axis shows the −log10 (*p*) values of the associations. Each point corresponds to a genetic marker positioned along the chromosomes, with higher −log10 (*p*) values indicating stronger marker–trait associations.Alternating colors (black and grey) distinguish adjacent chromosomes. The red horizontal line indicates the significance threshold at −log_10_ (*p*) = 3.0 (*p* ≤ 0.001).

Moreover, Manhattan plot analysis showed the statistical significance of the relationship between specific SNPs and the eight measured traits across all 185 HEB-25 genotypes (Figure 6). These signals were unevenly distributed along the genome across the traits, and several traits displayed distinct peaks exceeding the significance threshold. Under 40% seawater treatment, significant loci were detected associated with specific traits, including CL on chromosome 3H, RL and RSR on chromosomes 2H, 3H, 5H and 7H, Ger% on chromosomes 4H and 7H, FW on chromosomes 2H, 5H and 7H, DW on chromosomes 1H, 2H, 3H and 7H, and DW/FW_R on chromosomes 3H, 4H, 5H and 7H. Significant associations were detected for eight traits across all seven barley chromosomes (Figure 6). No significant tolerance index associations were identified under 40% seawater treatment compared with the control.

### 2.8. QTL Architecture Across HEB-25 Families

The GWAS analysis across all 185 HEB-25 genotypes from four families revealed several significant SNPs and QTLs associated with salinity stress tolerance. These loci were associated with biomass traits and stress tolerance indices, including QTLs for RLTI, CLTI, and Ger%TI. These QTLs highlight the advantage of the analysis across families in detecting loci with consistent effects across the full genetic diversity of the HEB-25 population.

### 2.9. Candidate Gene Identification for QTLs

The annotation of underlying candidate genes was categorized according to QTLs detected across all four HEB-25 families, as provided in Table 3.

The GWAS analysis across all HEB-25 families identified candidate genes underlying key QTLs associated with salinity stress tolerance, as provided in Table 3. Notably, the GWAS across HEB families identified QTLs underlying candidate genes which were not detected in individual family analyses such as QTL-CLTI-4H-1, QTL-CLTI-6H-1, QTL-GerTI-7H-1 and QTL-RLTI-2H-1) which encoding methionine S-methyltransferase (HORVU.MOREX.r2.1HG0002020), endonuclease/exonuclease/phosphatase family protein (HORVU.MOREX.r2.5HG0351360), calcium-dependent lipid-binding proteins (HORVU.MOREX.r2.7HG0531870), and NBS-LRR resistance protein (HORVU.MOREX.r2.1HG0003400) respectively in addition to other tolerance related QTLs highlighting the role of calcium signaling, biomass traits and cell elongation under seawater response. As well as combined QTLs harbored genes linked to epigenetic regulation, such as ATP-dependent Clp protease ATP-binding subunit (HORVU.MOREX.r2.7HG0532870), methyl-CpG-binding domain protein (HORVU.MOREX.r2.2HG0080660) as a chromatin-associated factor, suggesting conserved regulatory mechanisms. The direction of wild barley allelic effects at QTLs was interpreted based on additive effects relative to the Barke reference allele. Trait-increasing Hsp alleles showed positive additive effects, whereas trait-decreasing Hsp alleles showed negative additive effects, as provided in Table 3.

## 3. Discussion

Salinity tolerance in plants is a complex trait controlled by multi-genetic networks. To effectively improve crop resilience, a strategic approach must focus on identifying and stacking superior alleles within key stress-response genes. Therefore, pinpointing the specific genetic components and underlying genes is a fundamental prerequisite for breeding high-performance, salt-tolerant cultivars. In this study, four families from the NAM population HEB-25, comprising 185 genotypes, were evaluated under a 40% seawater treatment to assess their salinity stress tolerance index. In addition, a GWAS analysis was performed to identify MTAs associated with salinity tolerance. Salinity stress significantly influences physiological traits. The results revealed differences in response to salinity tolerance among all traits (Table 1). Many studies on barley have shown that salinity stress negatively affects physiological characteristics at different stages of plant growth and development [35,36].

In our study, salinity imposed by 40% seawater resulted in pronounced genotypic differences in germination and seedling stage responses among the four selected HEB-25 families. Family 01 displayed the highest TI values for coleoptile length (CL), fresh weight (FW), dry weight (DW), and germination percentage (Ger%), indicating a relatively strong maintenance of physiological productivity and biomass under salinity stress conditions. In contrast, Family 22 exhibited the lowest TI values for FW, DW, and the DW/FW ratio, suggesting an increased sensitivity of biomass accumulation to salinity. Moreover, family 04 showed the lowest TI values for Ger%, shoot length (ShL), and root length (RL) (Figure 1), reflecting impaired early establishment when exposed to high salinity. These patterns highlight the complex and trait-specific nature of salinity tolerance, where genotypes may perform differently across germination and seedling growth parameters. The differences in genotype distribution between control and salinity conditions reflect the multifaceted physiological responses triggered by salinity stress. Under saline conditions, plants experience both osmotic stress and ionic toxicity due to Na^+^ accumulation, disrupting K^+^/Na^+^ homeostasis and inhibiting cell elongation, which collectively explain the pronounced reductions in ShL and RL across genotypes [4,40]. The increased phenotypic differentiation under stress further reflects the genetic diversity within HEB-25, where genotypes carrying favorable wild donor alleles maintain relatively higher TI values through osmotic adjustment and antioxidant defense activation [41,42]. The use of tolerance indices has proven effective for screening barley germplasm under salinity stress, as it integrates performance under stress relative to control conditions and allows for ranking genotypes across multiple traits. Previous studies have similarly detected large variation among barley lines for salinity tolerance at the germination and seedling stages, reinforcing the value of TI metrics for identifying tolerant genotypes, e.g., in wild barley introgression lines under NaCl stress [43,44].

Salinity stress impacts germination and seedling growth primarily through osmotic effects and ion toxicity, which can inhibit water uptake and metabolic processes, leading to reduced shoot and root growth in sensitive genotypes [43,45]. Thus, the high TI values observed in family 01 may reflect superior physiological resilience, such as more efficient osmotic adjustment or ion exclusion, traits previously linked to salinity tolerance in barley and other cereals [46]. Interestingly, although family 04 exhibited low TI values for several individual traits, the overall tolerance index distribution suggested that family 04 showed the highest level of tolerance across the composite set of evaluated traits.

This suggests that some families may possess compensatory mechanisms allowing better overall stress coping strategies despite poor performance in specific traits, a phenomenon also noted in other complex stress response studies where salinity tolerance cannot be fully captured by single traits alone [47]. Collectively, these findings underline the importance of multi-trait evaluation and composite indices for robust salinity tolerance screening, particularly under extreme treatments like 40% seawater, which mimic severe saline environments expected under ongoing climate change and expanding soil salinization pressures [48].

GWAS was applied as a powerful new-generation sequencing tool to reveal the complex pathways of abiotic stress tolerance in barley [49,50]. Meanwhile, Cockram et al. [51] introduced GWAS to the barley genome and mapped candidate polymorphisms using 32 phenotypes and 15 morphological traits. GWAS has been successfully applied as a promising approach to defining the causative allele(s)/loci that can be used in breeding crops for adaptation to climate change. The approach for basic genetic and statistical concepts of GWAS and the explanation of how the candidate gene(s) for specific traits can be detected using bioinformatic tools was described by [46]. The present GWAS revealed a complex and highly polygenic genetic architecture underlying germination, seedling parameters and salinity tolerance in barley. The detection of 27 significant MTA and 20 QTLs under control, seawater and tolerance indices conditions demonstrates that the salinity response is regulated by multiple loci with environment and background-dependent effects. Similar polygenic control of salinity-related traits has been widely reported in barley [52,53,54].

The uneven distribution of QTLs across the seven barley chromosomes supports the deterministic genomic organization of stress-related loci. Chromosome 7H emerged as the most QTL-rich chromosome in the analysis across all HEB-25 families, bearing the highest number of QTLs and suggesting the presence of stable loci conserved across diverse genetic backgrounds. This agrees with [21,26] who identified chromosomes 4H, 5H and 7H as key salinity-responsive loci in barley during vegetative and reproductive stages in the NAM population. To conclude, chromosomes 4H and 7H have critical loci linked to salinity responses during the seedling, ripening and flowering stages, thereby reinforcing the relevance of these loci for breeding programs aimed at improving salinity tolerance across the developmental stages. The GWAS analysis across all HEB-25 families demonstrated increased power of QTL detection, particularly for tolerance index traits. Among the identified QTLs, RLTI, CLTI, and Ger%TI were exclusively detected in the across-families analysis, indicating that the number of discovered variants is strongly correlated with experimental sample size. This predicts that increasing sample size will further increase the number of discovered variants while capturing broader genetic diversity [55,56]. Notably, the across-families analysis was enriched with tolerance index traits, thereby supporting that tolerance indices are remarkable phenotypes for dissecting abiotic stress [57].

Candidate gene analysis revealed biologically meaningful pathways related to salinity tolerance and brought about beneficial information that would be used to improve salt tolerance in the given genotypes through genetic engineering or molecular breeding [58]. These genes include ion homeostasis, osmotic adjustment, hormone signaling, lipid remodeling, and transcriptional regulation. Among these gene families, i.e., histidine kinases, V-type proton ATPases, calcium-dependent lipid-binding proteins, aldehyde dehydrogenase and sugar transporters were identified. Their roles in salinity tolerance are mediated through multiple mechanisms, including stress signaling pathways [59], interaction of glycolytic enzymes with V-ATPase subunits [60], Ca^2+^ sensing and downstream salt-stress signal transduction [61], stress-responsive amino acid metabolism [62], as well as osmolyte regulation and ion transport processes [59]. NBS-LRR proteins, receptor-like kinases, and AP2/ERF transcription factors were enriched, suggesting that there may be cross-talk between salinity stress and resistance pathways [63,64,65]. That cross-talk between salt stress and pathogens can be found in cellular changes, responsive genes and downstream pathways, such as production of ROS, involvement of the same transcription factors and hormonal signaling pathways [64]. Importantly, the GWAS analysis across HEB-25 families identified candidate genes responsible for epigenetic regulation and plastid biogenesis, including methyl-CpG-binding proteins and ATP-dependent proteases [66,67]. Remarkably, epigenetic variation enhances plant plasticity by generating heritable phenotypes that improve fitness across generations [68]. Stable QTLs identified across families, especially on chromosome 7H, represent promising targets for marker-assisted selection and genomic selection in barley salinity tolerance breeding programs.

The QTL regions identified in the present study share genomic positions with loci previously mapped in the HEB-25 population under field salinity conditions. Saade et al. [33] conducted a GWAS on the full HEB-25 population of 1336 genotypes, focusing on yield-related traits including flowering time, harvest index and grain yield under saline irrigation. Despite the difference in the traits evaluated, including yield and agronomic parameters in Saade et al. versus germination and seedling growth parameters in the present study, several QTL positions converge on the same chromosomal regions, particularly on chromosomes 2H and 7H. A notable locus on chromosome 2H was associated with a favorable wild allele effect on yield under high salinity conditions. The detection of overlapping QTL regions across independent studies using different traits and phenotyping conditions strengthens the evidence for stable salinity-responsive loci in the HEB-25 population. This genomic consistency suggests that certain chromosomal regions harbor pleiotropic loci with broad effects on both early seedling development and later reproductive stages under salinity stress, making them particularly valuable targets for barley improvement programs.

Among the 20 QTLs identified across HEB-25 families, five loci demonstrated particularly large additive effects and high phenotypic variance explained (R^2^), making them the most promising candidates for marker-assisted selection in barley salinity breeding programs. The most prominent QTL was QTL-CLS-4H-1 on chromosome 4H, which explained the highest proportion of phenotypic variance for coleoptile length under salinity stress (R^2^ = 7.99%, a = +0.008). This chromosomal region is consistent with previous barley QTL mapping studies, where coleoptile length QTLs on chromosome 4H have been repeatedly identified under salt stress conditions [21]. The wild barley Hsp allele at this locus harbors a potassium transporter gene (HORVU.MOREX.r2.7HG0610980). Potassium transporters of the HKT family have been demonstrated to enhance salt tolerance in barley by reinforcing Na^+^/K^+^ homeostasis under ionic stress [69]. Introgression of this wild allele could therefore meaningfully improve coleoptile emergence and early seedling establishment under saline field conditions, where deep sowing is required to reach soil moisture. The second most influential QTL, QTL-FWS-7H-1 on chromosome 7H (R^2^ = 5.91%, a = −1.883), was associated with fresh weight under salinity stress. Chromosome 7H has previously been identified as a major genomic region controlling seedling growth responses to salinity in barley, with a major QTL for root elongation under salt stress detected on chromosome 7HS in the Nure × Tremois population [70], and multiple seedling fresh weight QTLs co-localizing on chromosome 7H [70]. The candidate gene at this locus encodes a calcium-dependent protein kinase (HORVU.MOREX.r2.1HG0055980). Calcium-dependent protein kinases are established mediators of Ca^2+^-triggered abiotic stress signaling in barley and other cereals [71]. The negative additive effect at this locus reflects a stress-adaptive strategy of biomass reallocation characteristic of tolerant genotypes under ionic stress.

Third, QTL-FWS-5H-1 on chromosome 5H (R^2^ = 4.05%, a = +1.496) was associated with fresh weight under salinity stress. Wild barley introgression studies have previously reported favorable exotic QTL alleles on chromosome 5H contributing to improved seedling biomass and root-to-shoot ratio under salinity stress [45]. The candidate gene at this locus encodes a mannosyltransferase (HORVU.MOREX.r2.2HG0153640), an enzyme involved in protein N-glycosylation. Protein glycosylation pathways in the endoplasmic reticulum are critical for maintaining cell wall integrity and cellulose biosynthesis under salinity conditions [72]. The positive additive effect of the wild Hsp allele at this locus suggests its capacity to maintain biomass accumulation under stress, which is directly relevant to yield stability.

Fourth, QTL-DWFWRS-4H-1 on chromosome 4H (R^2^ = 2.51%, a = +5.878) influenced the dry weight to fresh weight ratio under salinity stress. Chromosome 4H has been associated with multiple salinity-related biomass and physiological traits in barley [21,45], and the present study further confirms its importance for water use efficiency under ionic stress. The candidate gene at this locus encodes a methyl-CpG-binding domain protein (HORVU.MOREX.r2.2HG0080660), an epigenetic reader involved in chromatin-mediated regulation of stress-responsive gene expression [73]. The positive wild allele effect suggests that epigenetic fine-tuning of osmotic adjustment pathways may underlie tolerance at this locus.

Finally, QTL-RSRTI-2H-1 on chromosome 2H (R^2^ = 2.51%, a = +1.063) was associated with the root-to-shoot ratio tolerance index. Chromosome 2H has consistently emerged as a major hotspot for seedling-related QTLs in barley, with multiple studies reporting co-localized QTL clusters for shoot and root traits under salinity stress [45,74]. The candidate gene at this locus encodes villin (HORVU.MOREX.r2.3HG0272790), an actin-binding protein that regulates cytoskeletal dynamics and root hair growth in response to osmotic stress [5]. The positive wild Hsp allele effect at this QTL suggests an enhancement of root architecture plasticity under saline conditions, a key adaptive trait for salinity tolerance in barley.

While seedling-stage salinity tolerance provides valuable insights into genotypic variation, it does not necessarily predict performance at later developmental stages. However, early-stage tolerance remains critical for successful establishment under saline environments and provides an efficient platform for genetic dissection. Distinct mechanisms such as ion exclusion, Na^+^ compartmentalization, and restricted xylem loading become predominant [4,40]. Adult plant tolerance may therefore differ substantially from seedling responses, and the identified QTLs should be validated across multiple developmental stages and under field conditions to comprehensively assess their contribution throughout the barley life cycle [75]. Nevertheless, the seedling stage remains an efficient and reproducible platform for high-throughput genetic dissection of salinity tolerance, as evidenced by the significant QTLs and candidate genes identified in the present study.

Collectively, these five QTLs located on chromosomes 2H, 4H, 5H and 7H represent high-priority targets for marker-assisted selection and genomic selection in barley salinity breeding programs. Their chromosomal positions are supported by independent QTL mapping studies across diverse barley populations, underscoring their stability and potential for transfer into elite cultivars. Pyramiding these QTL alleles in a single elite background could lead to substantial cumulative improvements across germination, seedling growth and biomass traits under salinity stress. Based on the comprehensive TI analysis, HEB_22_003, HEB_04_087, and HEB_01_013 represent the most promising genotypes for salinity breeding, exhibiting the highest mean TI values across multiple traits and therefore constitute valuable germplasm for introgression into elite cultivars. Although the identified candidate genes provide solid genetic targets for salinity tolerance, further downstream functional validation, including qRT-PCR expression profiling, VIGS, and ectopic expression is highly recommended and represents an essential direction for our future investigations to fully elucidate their underlying molecular mechanisms.

## 4. Materials and Methods

### 4.1. Plant Materials

In this study, we used the parental genotypes of the HEB-25 population, Barke, and 25 wild barley accessions (24 wild barley *H. vulgare* ssp. *spontaneum* (Hsp) (HID_003, 005, 025, 027, 032, 033, 046, 055, 056, 064, 101, 106, 115, 127, 139, 144, 201, 213, 229, 246, 322, 352, 364, 371) and one Tibetan *H. vulgare* ssp. *agriocrithon* (Hag) (HID_380), as well as 185 selected BC1S3 lines from four selected HEB families (F 01 (56 genotypes), F 04 (42 genotypes), F 09 (52 genotypes), and F 22 (35 genotypes)) from the multi-parental barley NAM population Halle Exotic Barley (HEB-25) [28,76].

### 4.2. Genotyping of HEB-25 Families

DNA extraction and genotyping procedures are detailed in [39,77]. Briefly, DNA was extracted using the BioSprint 96 DNA Plant Kit (Qiagen, Hilden, Germany), then dissolved in distilled water to a concentration of approximately 50 ng/μL. Genotyping of the population was performed using the iSelect 50K chip at SGS Trait Genetics, Gatersleben, Germany [78]. A total of 32,995 SNPs were utilized in this study.

### 4.3. Growing Conditions, Germination and Seedling Traits

First, the parental genotypes (Barke and 25 wild barley accessions) were evaluated for stress adaptation traits under control (C) and salinity stress (S) treatments during germination and seedling stages. Accordingly, we selected four families based on the behavior of their wild parent (F 01, F 04, F 09, and F 22, including a total of 185 genotypes) to be evaluated at germination and seedling stages under salinity stress using 40% diluted seawater. The 40% seawater concentration was selected based on a preliminary screening of the parental genotypes, which identified this level as optimal for discriminating between tolerant and sensitive genotypes while maintaining sufficient phenotypic variation. This concentration corresponds to approximately 14.4 g L^−1^ total dissolved salts, equivalent to ~246 mM NaCl, falling within the range commonly applied in barley salinity studies (100–400 mM NaCl) [27]. Unlike pure NaCl solutions, diluted seawater provides a more ecologically relevant ionic environment, reflecting the complex salt composition encountered in saline agricultural soils and brackish irrigation water.

These experiments were conducted at the Leibniz Institute of Plant Genetics and Crop Plant Research (IPK), Gatersleben, Germany. For each genotype, sixteen seeds were surface-sterilized in 70% ethanol for 1 min, rinsed three times with sterile distilled water, and blotted dry on paper sheets (25 × 60 cm; Ahlstrom Munnksjö GmbH, Germany). Germination was performed following the International Seed Testing Association protocol ISTA 2014.

Seeds were positioned approximately 5 cm from the top edge of a pre-wetted paper sheet. The control treatment was irrigated with tap water, whereas the salinity treatment consisted of 40% diluted Mediterranean microfiltered seawater (Naturitas Essentials, Barcelona, Spain). The original seawater had a salt concentration of 36.0 g L^−1^ (pH 7.8) and contained 9.8 g L^−1^ Na, 23.2 g L^−1^ Cl, 3.4 g L^−1^ SO_4_, 0.359 g L^−1^ Mg, 0.465 g L^−1^ K, 0.409 g L^−1^ Ca, <2.4 mg L^−1^ B, and 0.144 g L^−1^ bicarbonates. Seeds were covered with a second wet sheet, carefully rolled to maintain separation, placed into plastic bags, and irrigated as required with the respective solutions.

Samples were incubated in a growth chamber (MLR-352-PE, Panasonic, Osaka, Japan) for 3 days at 20 ± 2 °C in complete darkness, followed by 7 days at 20 ± 2 °C (day) and 16 ± 2 °C (night) under a 16 h light/8 h dark photoperiod.

### 4.4. Assessment of Germination and Seedling Growth Parameters

After 10 days, germinated seeds (radicle length > 3 mm) were counted. Germination percentage (Ger%) was calculated as:

Ger% = (Number of germinated seeds/Total number of sown seeds) × 100

For each genotype, eight seedlings were randomly selected to measure shoot length (SL) and root length (RL) using a scaled ruler. The root-to-shoot ratio (RSR) was calculated as RL/SL. Fresh weight (FW) was measured using an ultra-micro balance (Sartorius AC 1215, Germany). Seedlings were then oven-dried at 60 °C for 72 h to determine dry weight (DW).

### 4.5. Stress Tolerance Index (STI)

To assess growth performance and evaluate genotypic variation in salinity tolerance, the Stress Tolerance Index (STI) was utilized across five key parameters. These included the Germination Percentage Tolerance Index (GPTI), Seedling Length Tolerance Index (SLTI), Seedling Fresh Weight Tolerance Index (SFWTI), Seedling Dry Weight Tolerance Index (SDWTI), and Water Content Percentage Tolerance Index (WCPTI). The STI values for each trait were determined following [79]:

STI = Trait value under salinity treatment/Trait value under control

### 4.6. ANOVA Analysis of Phenotypic Data

An analysis of variance (ANOVA) was performed to compare genotypes and traits using GENSTAT for Windows Ver. 19 (VSN International, Hemel Hempstead, UK) for germination and seedling growth traits. The significance level for ANOVA was set at *p* ≤ 0.05 to identify significant differences among genotypes (G), salinity treatments (T), and their interaction effects (G × T). Means were separated using Fisher’s Least Significant Difference (LSD) at a 0.05 probability level. Broad-sense heritability (H^2^) estimates were calculated following [80]:

$$H_{b}=\frac{\sigma_{g}^{2}}{\sigma_{p}^{2}},\;\;\;\;\;\;\;\;\;\;\;\;\;\;\;\sigma_{p}^{2}=(\sigma_{g}^{2})+\left(\frac{\sigma_{e}^{2}}{r}\right)$$

where $\sigma_{g}^{2}$ is genotypic variance, $\sigma_{p}^{2}$ is phenotypic variance, $\sigma_{e}^{2}$ is pooled error variance, and r is the number of up to 16 replicates.

Data visualization, including box plots and PCA representations, was performed using Python (version 3.12.12) in the Google Colab environment, employing NumPy (v2.0.2), Pandas (v2.2.2), and Matplotlib (v3.10.0) libraries [81]. For the restricted maximum likelihood (REML) analysis, Best Linear Unbiased Estimators (BLUEs) for each treatment were computed using the nlme package (v3.1-164) in R (v4.3.1) [82].

### 4.7. Genome-Wide Association Study (GWAS) and Candidate Gene Detection

GWAS was implemented to associate SNP markers with the recorded phenotypic traits. It was carried out with a total number of 32,995 polymorphic SNPs in an IBS IBD matrix (matrix ED) as stated by Maurer and Pillen [83] across the four HEB families, following a marker regression model. For this, all traits were regressed on the quantitative SNP marker scores obtained from the IBD genotype matrix. For this purpose, PROC GLM was used to fit the model:

y = μ + Marker + e

where y = observed phenotype, μ = intercept, Marker = effect of SNP marker, e = residual/error. Subsequently, single marker *p*-values resulting from an F-test (full model versus reduced model without marker effect), as well as R^2^ of the respective regression, were recorded. Marker effects were multiplied by 2 (to represent the absolute phenotypic difference between the homozygous wild barley and the homozygous Barke allele classes of SNP, according to the definition of the genotype scores). As the additive SNP marker effect corresponds to the slope of the regression line, its sign indicates whether the allele increases or decreases the phenotype. Based on the initial definition of the genotype matrix, positive slopes can therefore be interpreted as trait-increasing wild allele effects, while negative slopes represent trait-decreasing wild allele effects. *p*-values were adjusted for multiple testing using the Bonferroni-Holm procedure [84]. Population structure and genetic relatedness in the HEB-25 NAM population are inherently controlled by the family-based IBD genotype matrix, which captures within-family relationships through the shared recurrent parent (cv. Barke), thereby reducing the confounding effects typically addressed by Q + K models in diverse association panels [28,83].

The physical position of QTLs and highly confident candidate genes was defined using the barley database BARLEX of Morex version 2 [85]. The candidate region of a QTL was defined as 1.7 Mb windows surrounding each significant SNP. This threshold is consistent with previous GWAS in barley employing similar populations and genotyping platforms, which report linkage disequilibrium (LD) decay ranging from approximately 200 kb to up to 1 Mb, depending on the genomic region and recombination landscape [28,36]. The Barley database was used for molecular and cellular characterization and gene annotations of the candidate genes (https://apex.ipk-gatersleben.de/apex/f?p=284:10 (accessed on 2 January 2026)).

## 5. Conclusions

Our study demonstrates that significant genetic variation for salinity tolerance exists within the barley HEB-25 population, particularly at the germination and seedling stages. The integration of phenotypic screening, tolerance indices, and GWAS enabled the identification of 20 QTLs and several strong candidate genes associated with early-stage salt tolerance. Among these, five major-effect QTLs located on chromosomes 2H, 4H, 5H, and 7H showed the largest additive effects and explained phenotypic variance, highlighting them as the most promising targets for marker-assisted selection and genomic-assisted breeding. These loci are associated with key adaptive processes, including Na^+^/K^+^ homeostasis, Ca^2+^-dependent stress signaling, protein glycosylation, epigenetic regulation, and root architecture plasticity, all of which contribute to improved seedling establishment and biomass maintenance under saline conditions. Family 01 emerged as the most comprehensively tolerant family, whereas Family 04 displayed a contrasting dual profile sensitive at the family-mean level for early establishment traits, yet harboring outstanding individual genotypes with superior tolerance, together providing a valuable genetic framework for functional validation of salinity tolerance alleles. The consistency of the major QTLs with previously reported genomic hotspots further supports their stability and transferability into elite barley backgrounds. Pyramiding these favorable alleles has strong potential to generate cumulative gains in germination, seedling vigor, and biomass performance under salinity stress. Overall, these findings enhance our understanding of the genetic basis of barley salt tolerance and provide robust molecular resources for developing climate-resilient cultivars adapted to arid and semi-arid environments.

## Figures and Tables

**Table 1:** Estimation of the minimum (Min), maximum (Max), mean, standard deviation (SD), coefficient of variation (CV) and Reduction percentage (R%) of the studied traits under 40% seawater (Salinity) and under control conditions.

Traits	Treatment	Min	Max	Mean	SD	CV %	R%
Ger	Control	37.50	100	88.655	13.249	14.939	−8.2%
Ger	Salinity	25.00	100	81.386	15.626	19.200	
CL	Control	1.60	4.84	3.854	0.398	10.315	−10.9%
CL	Salinity	1.48	1.59	3.434	0.590	17.187	
ShL	Control	1.60	20.93	14.936	2.819	18.876	−67.9%
ShL	Salinity	1.50	7.65	4.788	1.144	23.898	
RL	Control	1.63	22.64	14.465	3.188	22.037	−64.5%
RL	Salinity	2.30	9.04	5.139	1.303	25.355	
RSR	Control	0.59	1.35	0.973	0.143	14.683	+13.2%
RSR	Salinity	0.55	2.21	1.101	0.259	23.511	
FW	Control	1.90	0.57	0.38	0.069	17.932	−50.0%
FW	Salinity	0.11	0.28	0.19	0.031	16.642	
DW	Control	0.02	0.07	0.039	0.008	18.799	+10.3%
DW	Salinity	0.02	0.06	0.043	0.007	16.690	
DW/FW_R	Control	0.06	0.19	0.11	0.018	16.985	+116.4%
DW/FW_R	Salinity	0.14	0.44	0.238	0.048	20.333	

**Table 2:** Total number of QTLs (T-QTLs) detected in the study under control, salinity and tolerance index.

Traits	No. T-QTLs	QTLs Under Control	QTLs Under Seawater	QTLs on chr. 1H	QTLs on chr. 2H	QTLs on chr. 3H	QTLs on chr. 4H	QTLs on chr. 5H	QTLs on chr. 6H	QTLs on chr. 7H
Ger%	1	1	0	0	0	0	0	0	0	1
DW/FW_R	4	1	3	0	1	0	1	0	1	0
FW	2	0	2	0	0	0	0	1	0	1
CL	1	0	1	0	0	0	1	0	0	0
RL	1	0	1	1	0	0	0	0	0	0
DW	1	0	1	0	0	0	0	1	0	0
FWTI	3	NA	NA	1	0	0	0	0	0	2
RLTI	1	NA	NA	0	1	0	0	0	0	0
RSRTI	3	NA	NA	0	1	1	0	0	1	0
CLTI	2	NA	NA	0	0	0	1	0	1	0
Ger%TI	1	NA	NA	0	0	0	0	0	0	1
Total	49	2	8	2	3	1	2	2	3	5

Ger%: germination percent; DW: Dry Weight; RL: Root Length; FW: Fresh Weight; DW/FW_R: DW/FW ratio; RSR: Root Shoot Ratio; CL: Coleoptile length; TI: tolerance index. NA: not applicable.

**Table 3:** List of 20 QTLs significantly associated with salinity stress traits in a GWAS across four HEB-25 families. Indicated are the chromosomal positions, physical intervals, MTA counts, potential candidate gene identification and annotation, and Hsp effect sizes of the QTLs.

QTL ID	Trait	Chromosome	Physical Interval (Mb)	MTA Count	MTA	Gene ID	Gene Annotation	Additive Effect of Hsp Allele	R^2^
QTL-CLTI-4H-1	CLTI	4H	4.85	1	JHI_Hv50k_2016_228054	HORVU.MOREX.r2.1HG0002020	Methionine S-methyltransferase	−4.91	1.50%
QTL-CLTI-6H-1	CLTI	6H	5.82	1	JHI_Hv50k_2016_370565	HORVU.MOREX.r2.5HG0351360	Endonuclease/exonuclease/phosphatase family protein	5.35	1.92%
QTL-CLS-4H-1	CLS	4H	601.57	1	JHI_Hv50k_2016_261641	HORVU.MOREX.r2.7HG0610980	Potassium transporter	0.01	7.99%
QTL-DWFWRC-1H-1	DW/FW_RC	1H	520.75	1	JHI_Hv50k_2016_45340	HORVU.MOREX.r2.2HG0143290	LINE-1 reverse transcriptase	−0.16	0.01%
QTL-DWFWRS-2H-1	DW/FW_RS	2H	11.32–11.39	2	SCRI_RS_177330	HORVU.MOREX.r2.7HG0532870	ATP-dependent Clp protease ATP-binding subunit	1.35	1.02%
					JHI_Hv50k_2016_64655	HORVU.MOREX.r2.2HG0083310	Disease resistance protein	1.35	1.02%
QTL-DWFWRS-4H-1		4H	5.27–5.28	2	JHI_Hv50k_2016_228163	HORVU.MOREX.r2.2HG0080660	Methyl-CpG-binding domain protein	5.88	2.51%
					JHI_Hv50k_2016_228164	HORVU.MOREX.r2.2HG0080660		5.88	2.51%
QTL-DWFWRS-6H-1		6H	30.81	1	JHI_Hv50k_2016_381520	HORVU.MOREX.r2.6HG0458630	protein kinase family protein	5.88	2.51%
QTL-DWS-5H-1	DWS	5H	24.46	1	SCRI_RS_175557	HORVU.MOREX.r2.6HG0457110	Wall-associated receptor kinase-like protein	−0.01	0.002%
QTL-FWTI-1H-1	FWTI	1H	11.66–11.67	2	JHI_Hv50k_2016_11221	HORVU.MOREX.r2.6HG0451680	Mannosyl transferase	−0.04	0.01%
					JHI_Hv50k_2016_11229	HORVU.MOREX.r2.6HG0451680		−0.04	0.01%
QTL-FWTI-7H-1		7H	34.6	1	JHI_Hv50k_2016_458557	HORVU.MOREX.r2.5HG0358800	Glucan 1,3-beta-glucosidase	−3.00	1.30%
QTL-FWTI-7H-2			35.2	1	JHI_Hv50k_2016_458804	HORVU.MOREX.r2.3HG0194300	Cellulose synthase, putative	−2.995	1.30%
QTL-FWS-5H-1	FWS	5H	585.26–585.70	3	SCRI_RS_161728	HORVU.MOREX.r2.5HG0441970	Receptor-like protein kinase	0.003	0.001%
					SCRI_RS_212523	HORVU.MOREX.r2.2HG0153640	Mannosyltransferase	1.50	4.05%
					SCRI_RS_197945	HORVU.MOREX.r2.2HG0153640		0.003	0.001%
QTL-FWS-7H-1		7H	451.63	1	SCRI_RS_115426	HORVU.MOREX.r2.1HG0055980	Calcium-dependent protein kinase	−1.88	5.91%
QTL-GerTI-7H-1	Ger%TI	7H	9.41	2	JHI_Hv50k_2016_444783	HORVU.MOREX.r2.1HG0004010	Retrotransposon protein, putative, Ty1-copia subclass	−6.23	1.23%
					JHI_Hv50k_2016_444797	HORVU.MOREX.r2.7HG0531870	Calcium-dependent lipid-binding domain-containing protein	−6.23	1.23%
QTL-GerC-7H-1	Ger%C	7H	15.83	1	JHI_Hv50k_2016_450461	HORVU.MOREX.r2.4HG0280690	Glutamyl-tRNA(Gln) amidotransferase subunit A	2.02	0.003%
QTL-RLTI-2H-1	RLTI	2H	8.04	1	JHI_Hv50k_2016_62968	HORVU.MOREX.r2.1HG0003400	Disease resistance protein (NBS-LRR class) family	−0.04	0.006%
QTL-RLS-1H-1	RLS	1H	554.71	1	SCRI_RS_176006	HORVU.MOREX.r2.7HG0600630	Serine/threonine-protein kinase	0.004	1.67%
QTL-RSRTI-2H-1	RSRTI	2H	620.23	1	JHI_Hv50k_2016_102148	HORVU.MOREX.r2.3HG0272790	Villin	1.06	2.51%
QTL-RSRTI-3H-1		3H	1.81	1	JHI_Hv50k_2016_149604	HORVU.MOREX.r2.5HG0349560	Fantastic four-like protein	8.23	1.64%
QTL-RSRTI-6H-1		6H	47.260–47.264	2	BOPA1_4191_268	HORVU.MOREX.r2.6HG0462000	Plant protein 1589 of unknown function	−3.04	1.98%
					BOPA2_12_20381	HORVU.MOREX.r2.6HG0462000		−3.04	1.982%

## Data Availability

The data presented in this study are available on request from the corresponding author.

## Supplementary Materials

The following supporting information can be downloaded at: https://www.mdpi.com/article/10.3390/plants15121886/s1; Figure S1: Variation in five salinity tolerance indices (STIs) across 25 HEB families derived from distinct wild barley donors, evaluated against the commercial cultivar ‘Barke’ under salinity stress. Families 01 to 24 correspond sequentially to the *H. vulgare* ssp. *spontaneum* (Hsp) donors (HID_003, 005, 025, 027, 032, 033, 046, 055, 056, 064, 101, 106, 115, 127, 139, 144, 201, 213, 229, 246, 322, 352, 364, and 371), while Family 25 corresponds to the Tibetan *H. vulgare* ssp. *agriocrithon* (Hag) donor (HID_380). ‘Barke’ is included at the end of each panel as the recurrent parent/control. Red boxes highlight the most promising wild-donor-derived families exhibiting superior salinity tolerance indices compared to Barke (Family 01, Family 04, Family 09, and Family 22). Asterisks * indicate statistically significant differences compared to ‘Barke’ at *p* < 0.05 and *p* < 0.01, respectively. Figure S2: The boxplot analysis for phenotypic response to salinity stress for the eight traits. (A) Family 01; (B) Family 04; (C) Family 09; (D) Family 22. Figure S3: Phenotypic Correlation Heatmap (heatmap for family 01 genotypes). The degree of significance for all correlations was *p* ≤ 0.001. The color reflects the strength of the correlation. Figure S4: Phenotypic Correlation Heatmap (heatmap for family 04 genotypes). The degree of significance for all correlations was *p* ≤ 0.001. The color reflects the strength of the correlation. Figure S5: Phenotypic Correlation Heatmap (heatmap for family 09 genotypes). The degree of significance for all correlations was *p* ≤ 0.001. The color reflects the strength of the correlation; Figure S6. Phenotypic Correlation Heatmap (heatmap for family 22 genotypes). The degree of significance for all correlations was *p* ≤ 0.001. The color reflects the strength of the correlation; Figure S7: Principal Component Analysis (PCA) Biplot. (A) Family 01; (B) Family 04; (C) Family 09; (D) Family 22. Table S1: Summary statistics of investigated traits under control and salinity conditions at germination and seedling stage in parental lines of the HEB-25 population. Table S2: Analysis of variance, coefficient of determination (R^2^) and broad-sense heritability for traits studied under control and salinity conditions at germination and seedling stages in the parental lines of the HEB-25 population. Table S3: ANOVA, broad-sense heritability, and summary statistics of different salinity tolerance indices. Table S4: The tolerance index (TI) for the eight traits in the four families. Table S5: List of QTLs associated with multiple traits in the HEB-25 tested genotypes analysis and the annotation of underlying candidate genes.
