# Supplementary material for: Decoding the Genetic Basis of Salinity Tolerance at Germination and Seedling Traits in HEB-25 Barley NAM Population
Source: Plants (Basel). 2026 Jun 17;15(12):1886. doi: 10.3390/plants15121886 (PMC13306622; doi:10.3390/plants15121886)
Supplement: Supplementary file 1 [file plants-15-01886-s001.zip › plants-4327647-supplementary.pdf]

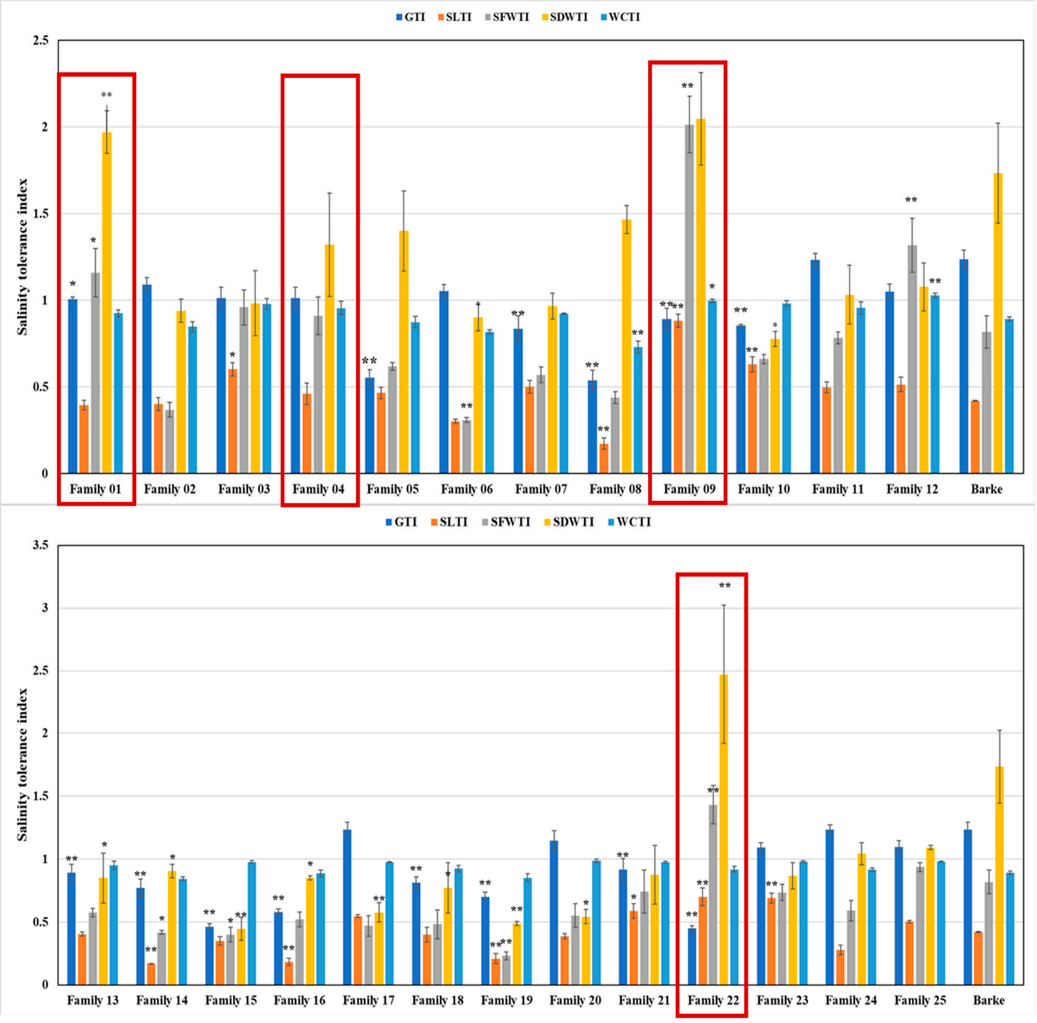

Figure S1. Variation of five salinity tolerance indices (STIs) across 25 HEB families derived from distinct wild barley donors, evaluated against the commercial cultivar 'Barke' under salinity stress. Families 01 to 24 correspond sequentially to the H. vulgare ssp. spontaneum (Hsp) donors (HID_003, 005, 025, 027, 032, 033, 046, 055, 056, 064, 101, 106, 115, 127, 139, 144, 201, 213, 229, 246, 322, 352, 364, and 371), while Family 25 corresponds to the Tibetan H. vulgare ssp. agriocrithon (Hag) donor (HID_380). 'Barke' is included at the end of each panel as the recurrent parent/control. Red boxes highlight the most promising wild-donor-derived families exhibiting superior salinity tolerance indices compared to Barke (Family 01, Family 04, Family 09, and Family 22). Asterisks *indicate statistically significant differences compared to 'Barke' at $p < 0.05$ and $p < 0.01$, respectively.

a) family 01

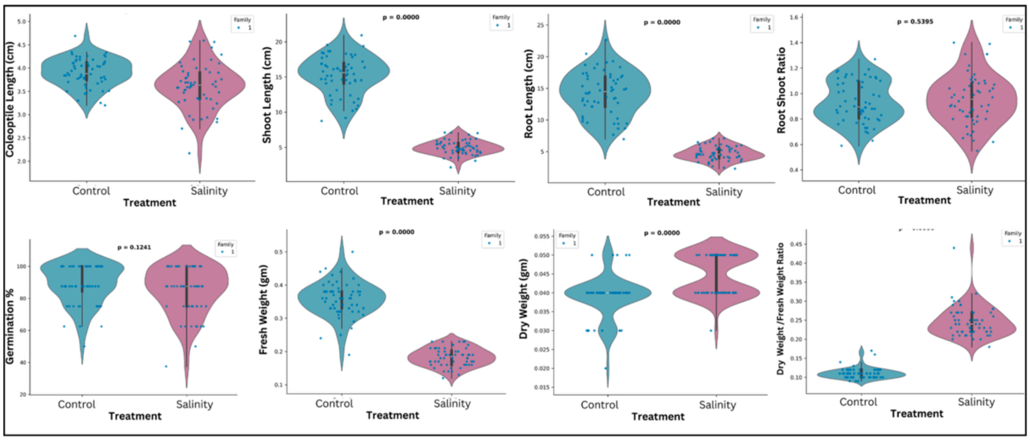

b) family 04

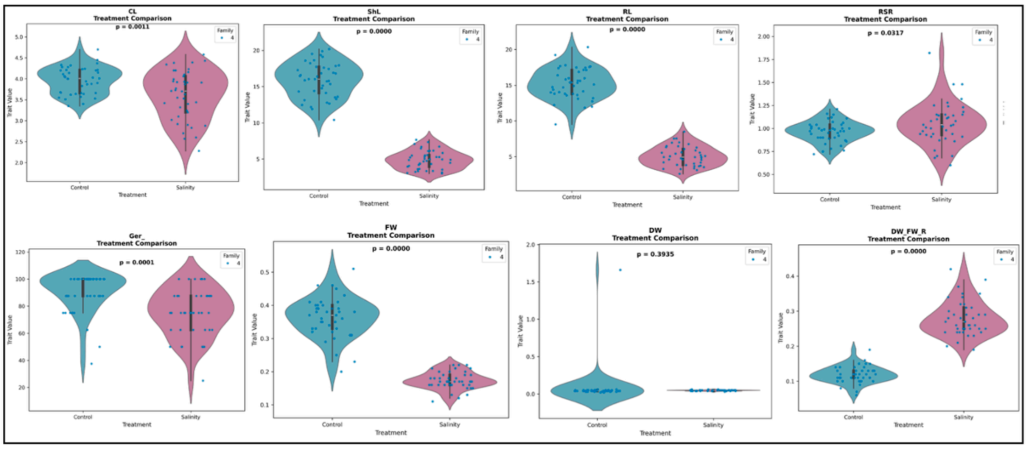

c) family 09

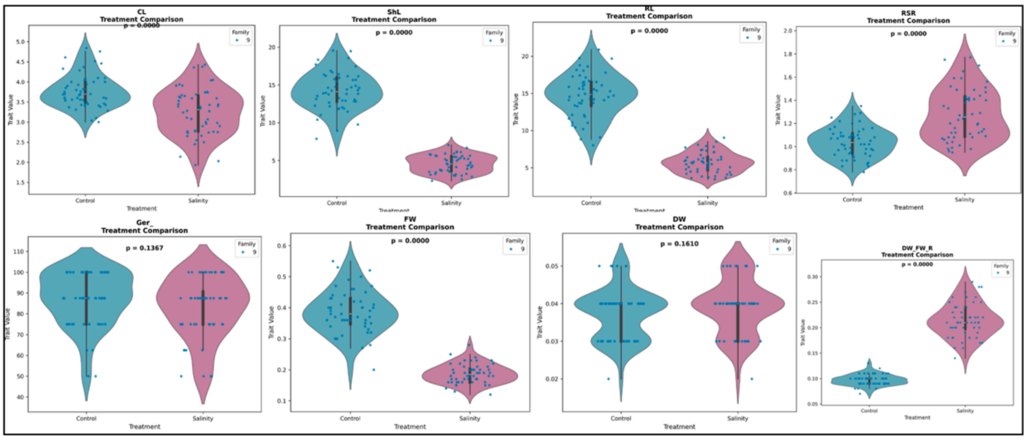

d) family 22

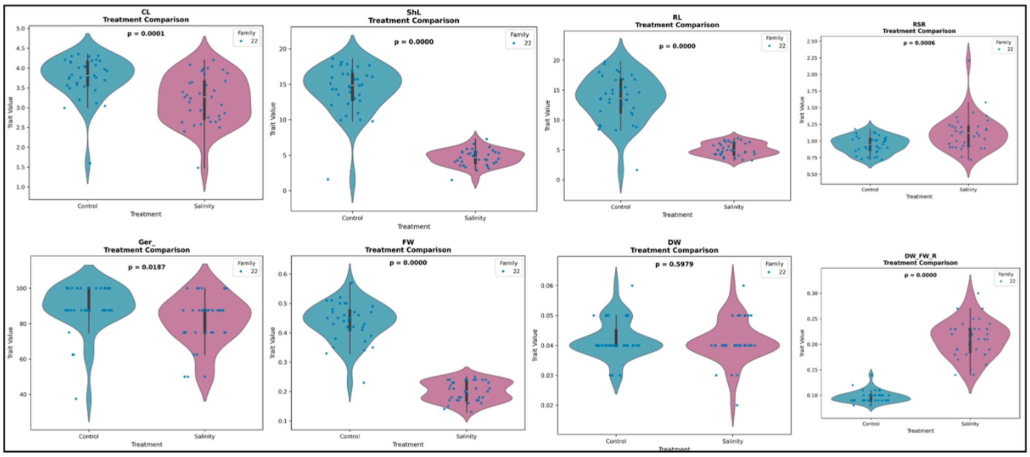

Figure S2: The boxplot analysis for phenotypic response to salinity stress for the eight traits. A) Family 01; B) Family 04; C) Family 09; D) Family 22.

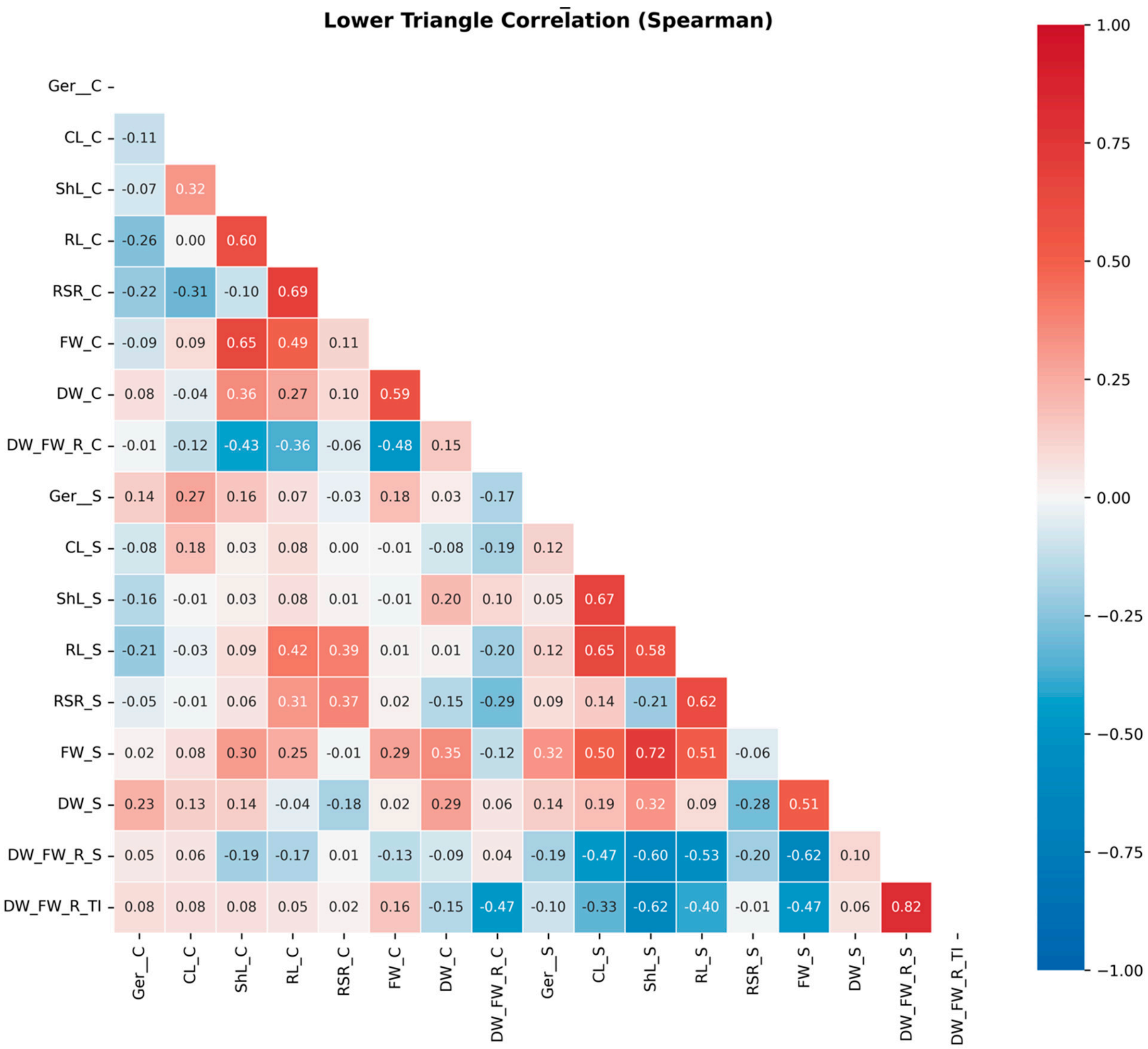

Figure S3. Phenotypic Correlation Heatmap (heatmap for family 01 genotypes). The degree of significance for all correlations was P≤0.001. The color reflects the strength of the correlation. Black crosses indicate non-significant correlations

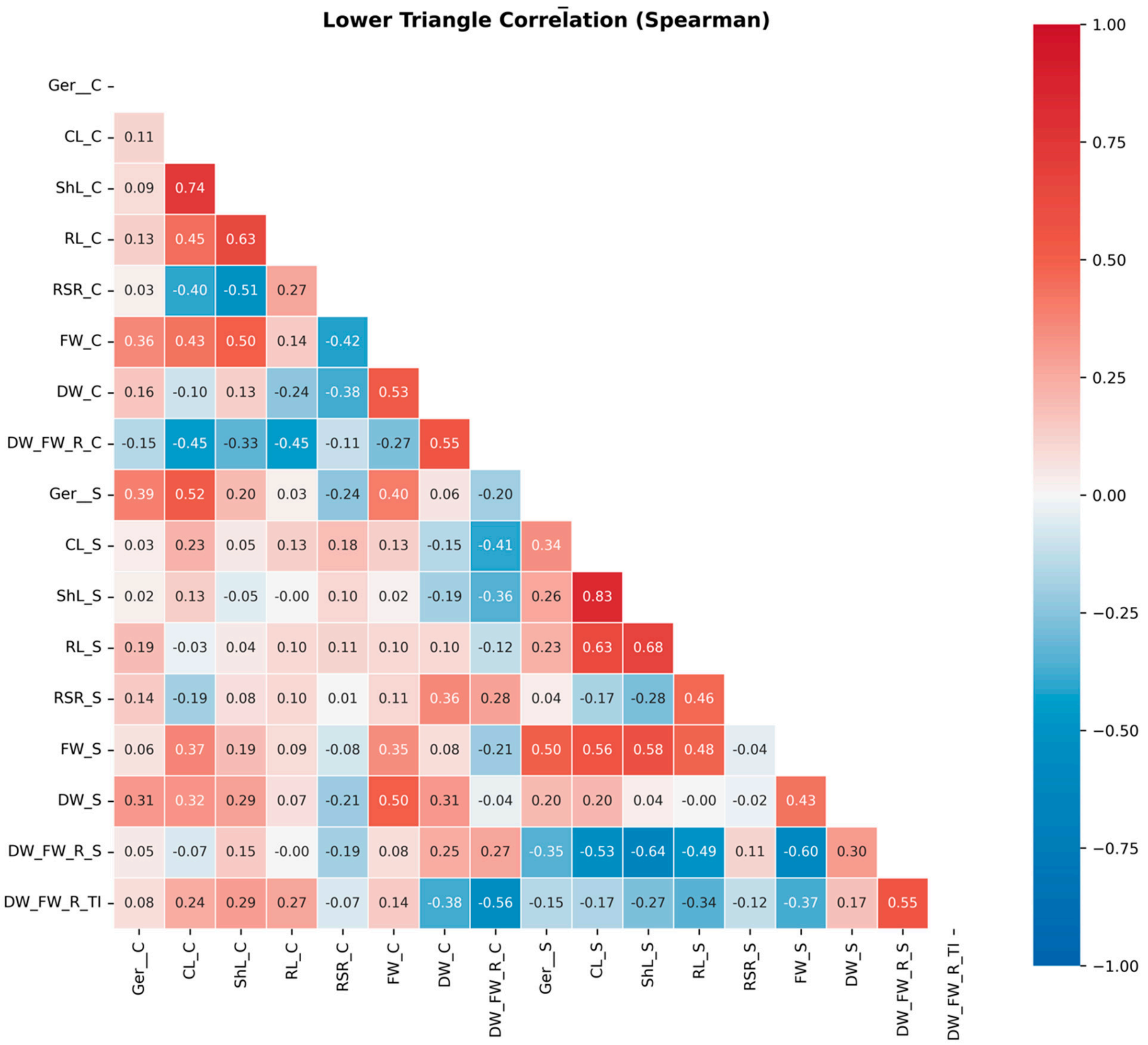

Figure S4. Phenotypic Correlation Heatmap (heatmap for family 04 genotypes). The degree of significance for all correlations was P≤0.001. The color reflects the strength of the correlation. Black crosses indicate non-significant correlations

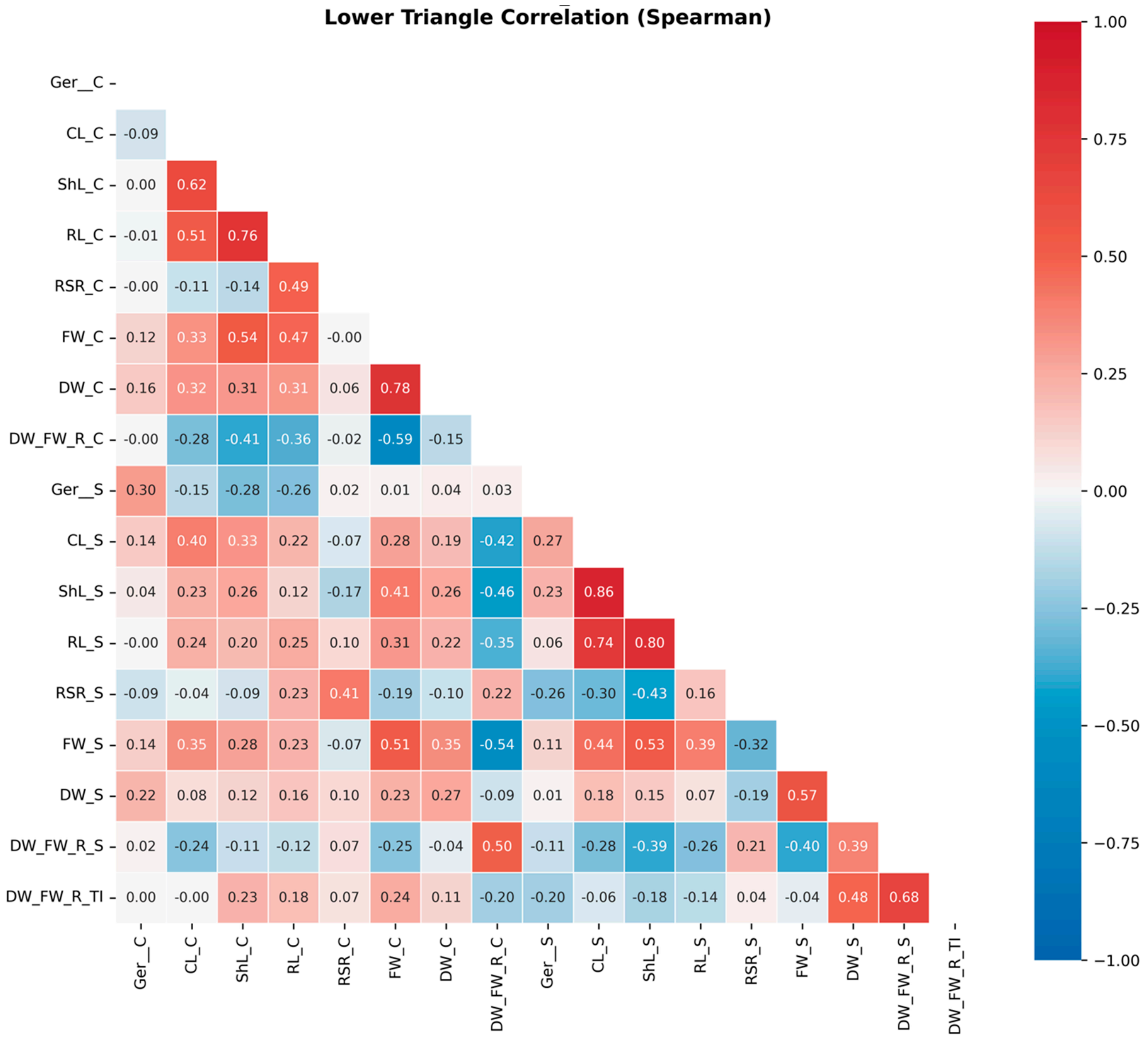

Figure S5. Phenotypic Correlation Heatmap (heatmap for family 09 genotypes). The degree of significance for all correlations was P≤0.001. The color reflects the strength of the correlation. Black crosses indicate non-significant correlations

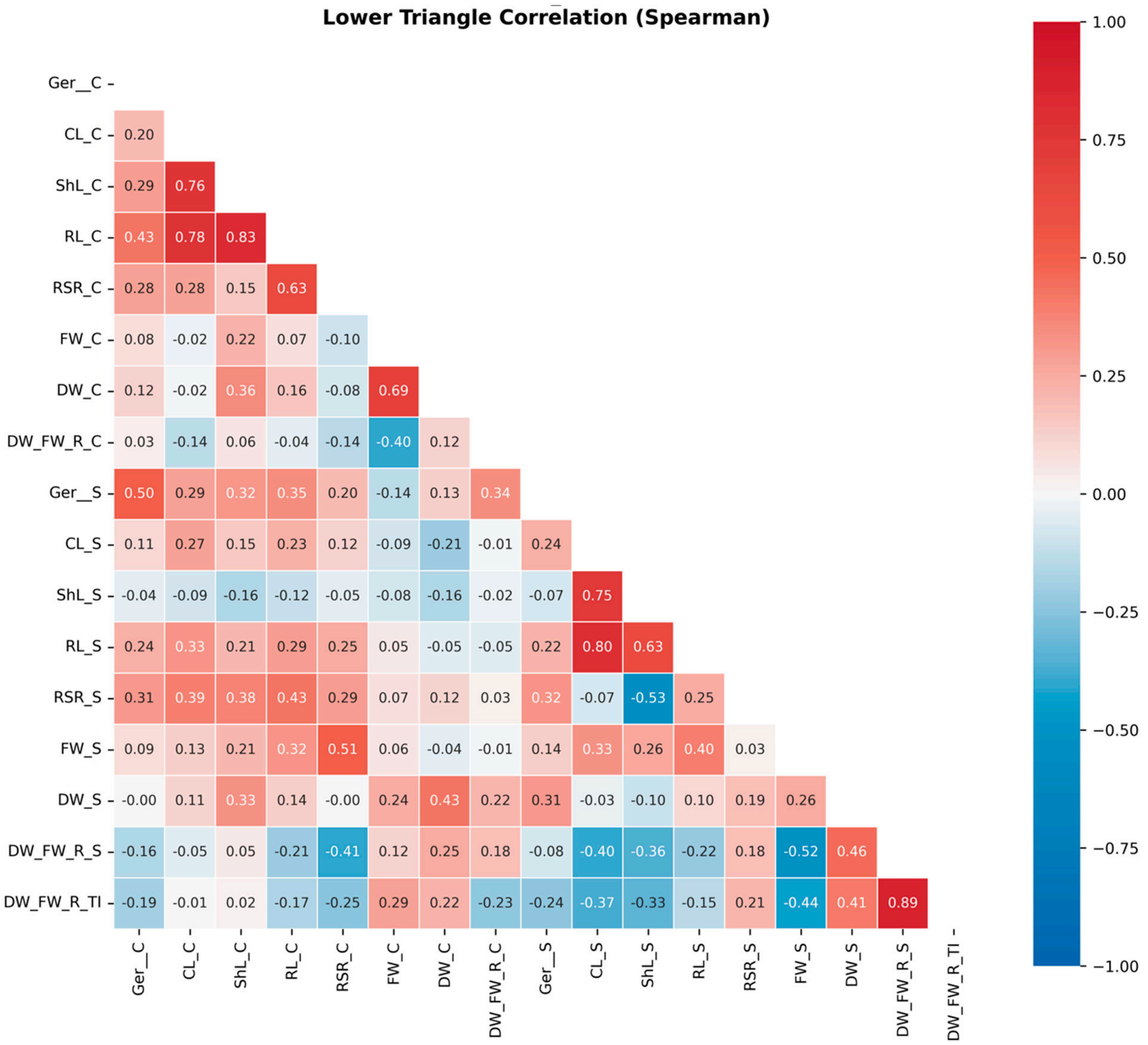

Figure S6. Phenotypic Correlation Heatmap (heatmap for family 22 genotypes). The degree of significance for all correlations was P≤0.001. The color reflects the strength of the correlation. Black crosses indicate non-significant correlations

a) family 01

b) family 04

c) family 09

d) family 22

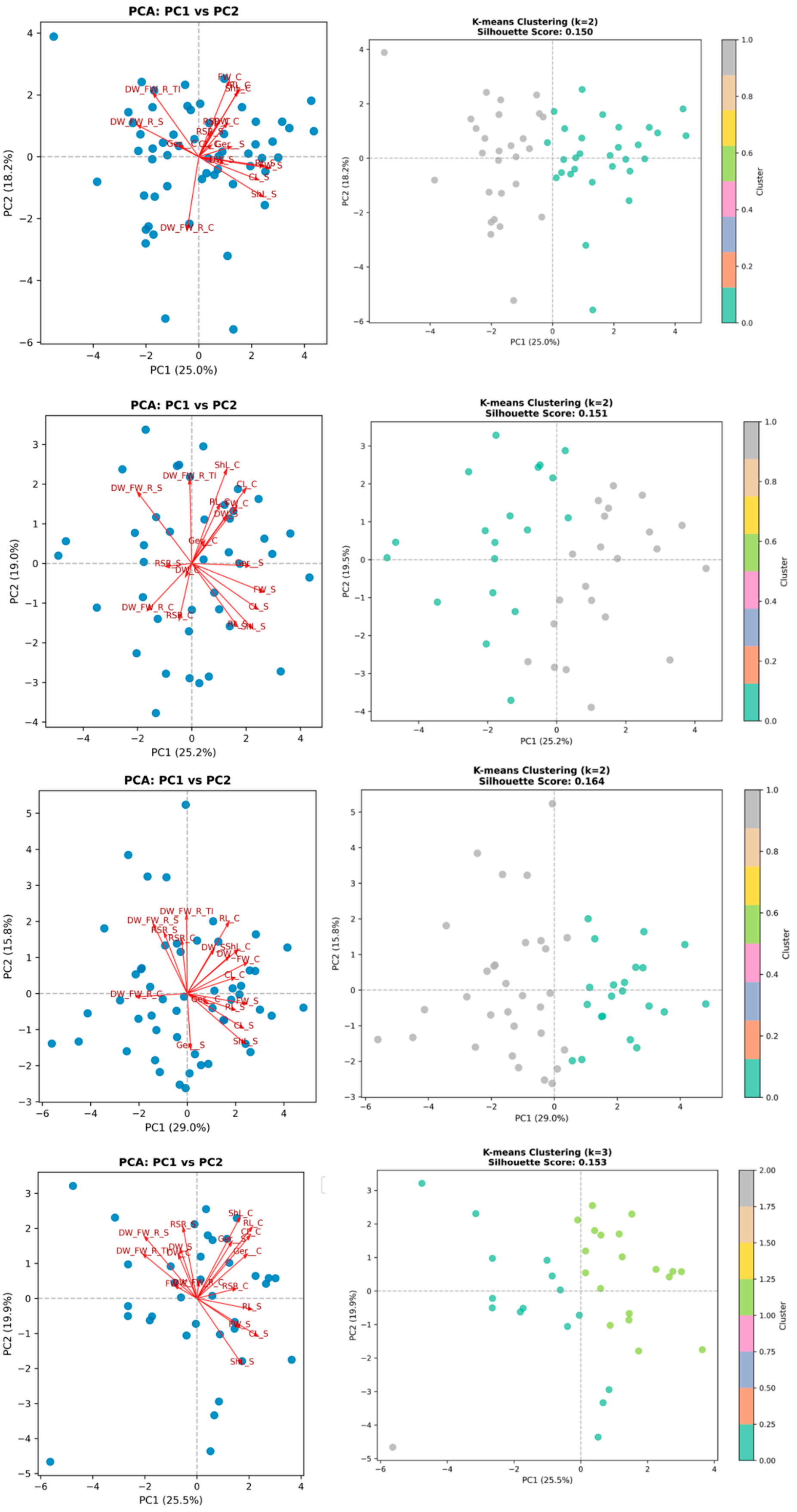

Figure S7. Principal Component Analysis (PCA) Biplot. A) Family 01; B) Family 04; C) Family 09; D) Family 22.
